# Hyperthyroid and Hypothyroid Status Was Strongly Associated with Gout and Weakly Associated with Hyperuricaemia

**DOI:** 10.1371/journal.pone.0114579

**Published:** 2014-12-08

**Authors:** Lai-Chu See, Chang-Fu Kuo, Kuang-Hui Yu, Shue-Fen Luo, I-Jun Chou, Yu-Shien Ko, Meng-Jiun Chiou, Jia-Rou Liu

**Affiliations:** 1 Department of Public Health, & Biostatistics Core Laboratory, Molecular Medicine Research Centre, Chang Gung University, Taoyuan, Taiwan; 2 Division of Rheumatology, Allergy and Immunology, Chang Gung Memorial Hospital, Taoyuan, Taiwan; 3 Division of Academic Rheumatology, School of Clinical Sciences, University of Nottingham, Nottingham, United Kingdom; 4 Department of Pediatrics, Chang Gung Memorial Hospital, Taoyuan, Taiwan; 5 Health Evaluation Center, Chang Gung Memorial Hospital, Taoyuan, Taiwan; 6 First Division of Cardiology, Chang Gung Memorial Hospital, Taoyuan, Taiwan; Johns Hopkins Bloomberg School of Public Health, United States of America

## Abstract

**Objectives:**

The aim of this study was to estimate the risk of hyperuricaemia and gout in people with hypothyroid or hyperthyroid status.

**Methods:**

This study analyzed data from individuals who participated in health screening programs at Chang Gung Memorial Hospital in northern Taiwan (2000–2010). Participants were categorized as having euthyroid, hypothyroid, or hyperthyroid status according to their thyroid-stimulating hormone (TSH) levels. Multinomial logistic regression models were used to calculate the odds ratios (95% CI) for hyperuricaemia and gout in participants with thyroid dysfunction compared to euthyroid participants.

**Results:**

A total of 87,813 (euthyroid, 83,502; hypothyroid, 1,460; hyperthyroid, 2,851) participants were included. The prevalence of hyperuricaemia was higher in hyperthyroid subjects (19.4%) than in euthyroid subjects (17.8%) but not in hypothyroid subjects (19.3%). The prevalence of gout was significantly higher in both hypothyroid (6.0%) and hyperthyroid (5.3%) subjects than in euthyroid subjects (4.3%). In men, hypothyroid or hyperthyroid status was not associated with hyperuricaemia. However, hypothyroid or hyperthyroid status was associated with ORs (95% CI) of 1.47 (1.10–1.97) and 1.37 (1.10–1.69), respectively, for gout. In women, hypothyroid status was not associated with hyperuricaemia or gout. However, hyperthyroid status was associated with ORs (95% CI) of 1.42 (1.24–1.62) for hyperuricaemia and 2.13 (1.58–2.87) for gout.

**Conclusions:**

Both hyperthyroid and hypothyroid status were significantly associated with gout and weakly associated with hyperuricaemia. A thyroid function test for gout patients may by warranted.

## Introduction

Gout is the most common inflammatory arthritis caused by hyperuricaemia and monosodium urate crystal deposition in joints and surrounding tissues [1]. In addition to recurrent acute arthritis, subcutaneous tophi and chronic painful arthritis [2], it also associates with multiple comorbidities [3]–[5] and increased mortality [6]–[8]. In addition to hyperuricaemia, other risk factors include male gender [9], increased age [9], [10], obesity [11], high purine and alcohol intake [12], chronic renal impairment [13] and the use of diuretics and other drugs [14], [15].

Few data are available on the association between thyroid dysfunction anduric acid metabolism and the evidence is conflicting. The correlation between TSH and serum uric acid levels was weak [16], [17]. However, previous studies have reported high prevalence of hyperuricaemia in patients with both hypothyroidism [18] and hyperthyroidism [18], [19]. A possible explanation for these potential associations is the ability of thyroid hormone to influence serum urate levels through regulation of glomerular filtration rate [20]. In contrast, only several older case series reported an association between gout and hypothyroidism [21]–[23] and the association between hyperthyroidism and gout has not been reported.

Therefore, using data from a large cohort consisting of healthy individuals who underwent health screenings, this study aimed to estimate the risk of hyperuricaemia and gout in people with thyroid dysfunction (both hypothyroid and hyperthyroid status).

## Methods

### Study population

This study used anonymised data therefore is exempted from patient consents, which procedure was approved by the Institutional Review Board of the Chang Gung Memorial Hospital (CGMH) (approval number: 97–1564C). Participants in a health-screening program at Chang Gung Memorial Hospital from 2000 to 2010 were enrolled in this study. Participants without tests for thyroid-stimulating hormone and serum uric acid levels were excluded.

### Health screening program procedures

Individuals participating in the health-screening program were assessed by physicians at the Health Promotion Centre in the Taoyuan branch of CGMH. Participants were asked to fast for 8 hours overnight before the date of screening. A structured questionnaire was filled out by each participant, which details personal information, diet, and medical history. A trained nurse recorded body weight, height, and sitting blood pressure after a 15-minute rest. A blood sample was taken at approximately 9:00 in the morning. Afterwards, participants received a series of examinations, including an electrocardiogram, chest and abdominal plain film, specialist consultations, and other selected examinations such as liver and heart echocardiography, gastrointestinal endoscopy, pulse wave velocity for arterial stiffness and coronary calcium scores. A physician performed all history taking and clinical examinations and gave an overall conclusion based on the procedure results.

### Measurements and classifications

All blood specimens were sent to the clinical laboratory at CGMH. External quality control for laboratory data was provided through participation in an international program implemented by the College of American Pathologists, as well as the National Quality-Control Program in Taiwan.

In the present study, subjects were grouped into normouricaemia, hyperuricaemia, and gout, which were treated as outcome variables. Hyperuricaemia was defined as a serum uric acid level above 7.7 mg/dL in men or above 6.6 mg/dL in women [24]. In this study, gout was defined as self-reported physician-diagnosed gout or a physician record of diagnosis of gout with an ICD-9 (International Classification of Diseases, Ninths revision) code of 274.0 or 274.9 in the outpatient department of CGMH. The validity of self-reported physician-diagnosed gout has been demonstrated in a previous study [25]. All participants were categorized by their thyroid stimulating hormone (TSH) levels as having euthyroid (TSH 0.35–5.5 µIU/mL), hypothyroid (TSH>5.5 µIU/mL), or hyperthyroid (TSH <0.35 µIU/mL) status.

Estimated glomerular filtration rate (eGFR) was calculated using the Modification of Diet in Renal Disease (MDRD) equation [26] as follows: eGFR  = 170× sCr^−0.999^ × Age^−0.176^ × BUN^−0.170^ × Alb^+0.318^ ×0.762 (if female). Low eGFR was defined as having an eGFR lower than 60 mL/min/1.73 m^2^ [27].

Metabolic syndrome was defined using the original Adult Treatment Panel III (ATP III) criteria and the modified ATP III criteria, in which waist circumference measurement was replaced by a body mass index (BMI)>27 kg/m^2^, according to previously modified criteria endorsed by the Bureau of Health Promotion in Taiwan [28], [29]. Therefore, for a diagnosis of metabolic syndrome in this study, 3 or more of the following criteria should be present: BMI>27 kg/m^2^, blood pressure ≥130/85 mmHg, high-density lipoprotein (HDL)-cholesterol level <40 mg/dL (1.04 mmol/L) for men or <50 mg/dL (1.30 mmol/L) for women, triglyceride level ≥150 mg/dL (1.70 mmol/L), and fasting glucose level ≥110 mg/dL (6.1 mmol/L).

### Statistical Analysis

For the primary analysis, we used the number of subjects as the denominator. Cases of hyperuricaemia and gout were identified according to the aforementioned criteria. The crude prevalences of hyperuricaemia and gout were calculated for the euthyroid, hypothyroid, and hyperthyroid groups. Using the euthyroid group as reference, the differences in baseline characteristics were tested using the Student's *t* test for continuous data and chi-square test for categorical data. Multinomial logistic regression was used to determine the factors affecting the risk of hyperuricaemia and gout. Multivariate relative risks (RRs) were adjusted for age, thyroid function status, the presence of low eGFR, and the number of metabolic syndrome components. Analyses were conducted separately for men and women. A two-sided p value ≤0.05 was considered statistically significant. All analyses were performed using IBM SPSS statistics software, version 19.0.

## Results

From 2000 to 2010, a total of 87,813 people were enrolled in our study. The prevalence of hypothyroid and hyperthyroid status were 1.7% (men, 1.1%; women, 2.3%) and 3.2% (men, 2.4%; women, 4.2%), respectively. Baseline characteristics stratified by thyroid status are shown in Table 1. Mean serum levels of uric acid did not differ among euthyroid, hypothyroid, and hyperthyroid subjects. The overall prevalence of hyperuricaemia and gout were 17.9% (men, 20.4%; women, 14.8%) and 4.4% (men, 6.5%; women, 1.6%), respectively. Hyperthyroid, but not hypothyroid individuals had significantly higher prevalence of hyperuricaemia. However, both hypothyroid and hyperthyroid subjects had significantly higher prevalence of gout than euthyroid subjects.

**Table 1:** Baseline characteristics of euthyroid, hyperthyroid and hypothyroid study subjects.

Variables	Euthyroid	Hypothyroid	Hyperthyroid
Number of subjects	83,502	1,460	2,851
Men (n (%))	47,017 (56.3)	545 (37.3)*	1,191 (41.8)*
Age (years)^a^	48.9±13.0	53.0±12.8*	52.3±14.2*
Uric acid (mg/dL)^a^	6.1±1.6	6.0±1.8	6.0±1.7
Hyperuricaemia (n (%))	14,895 (17.8)	282 (19.3)	552 (19.4)*
Gout (n (%))	3,579 (4.3)	87 (6.0)*	158 (5.5)*
Low eGFR (n (%))	4,192 (5.0)	170 (11.7)*	192 (6.7)*
Metabolic syndrome (n (%))	26,293 (18.2)	339 (23.2)*	467 (16.4)*
Obesity (n (%))	17,715 (21.4)	349 (24.3)*	487 (17.3)*
Hypertension (n (%))	38,838 (46.5)	720 (49.3)*	1362 (47.8)
Low high-density lipoprotein (n (%))	19,383 (23.2)	423 (29.0)*	837 (29.4)*
Hypertriglyceridaemia (n (%))	23,797 (28.5)	518 (35.5)*	597 (20.9)*
Fasting hyperglycaemia (n (%))	11,107 (13.3)	204 (14.0)	489 (17.2)*

* p<0.05, significant different from participants with euthyroid; ^a^ expressed as mean ± standard deviation.

There was no significant correlation between TSH and serum uric acid levels, with a correlation coefficient of −0.005 (p = 0.164). However, if analyzed separately by gender, there was a positive, but very weak, correlation between TSH and serum uric acid levels in men (correlation coefficient, 0.018; p<0.001) but not in women (correlation coefficient, 0.003; p = 0.531).

**Figure 1:**
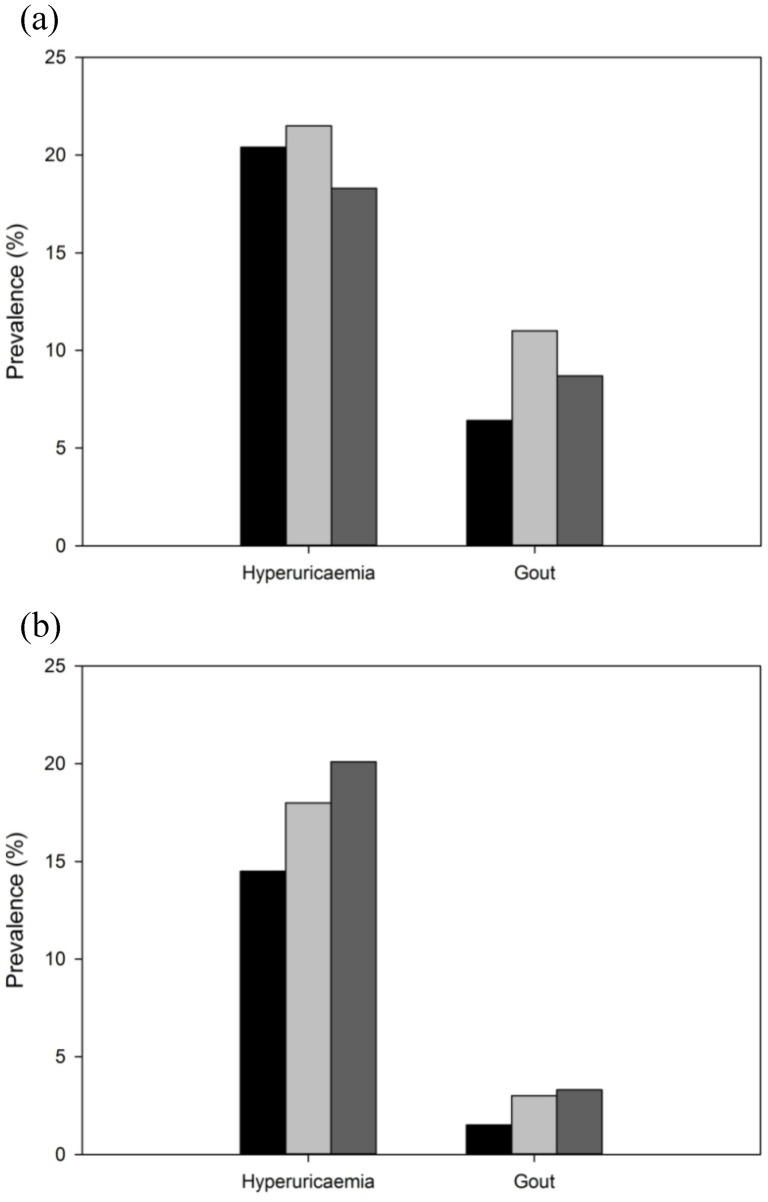
Prevalence of hyperuricaemia and gout in men (a) and women (b) with euthyroid (black bar), hypothyroid (light gray bar), and hyperthyroid status (dark gray bar).

Fig. 1 shows the sex-specific prevalence of hyperuricaemia and gout in euthyroid, hypothyroid, and hyperthyroid individuals. In men, the prevalence of hyperuricaemia in hypothyroid and hyperthyroid subjects did not differ significantly from the prevalence in euthyroid subjects; however, the prevalence of gout was significantly higher in hypothyroid and hyperthyroid individuals than in euthyroid individuals. In women, the prevalence of gout and hyperuricaemia were significantly higher in both hypothyroid and hyperthyroid individuals than in euthyroid individuals.

Next, we performed multinomial logistic regression to determine the factors associated with risk of hyperuricaemia or gout. Table 2 and Table 3 show the results in men and women, respectively. In general, low eGFR and the number of metabolic syndrome components were risk factors for hyperuricaemia and gout, and these factors had a stronger association than thyroid dysfunction. In men, neither hypothyroid status nor hyperthyroid status was associated with hyperuricaemia. Hypothyroid and hyperthyroid statuses were associated with ORs (95% CIs) of 1.47 (1.10–1.97) and 1.37 (1.10–1.69) for gout, respectively. In women, hypothyroid status was not associated with either hyperuricaemia or gout. However, hyperthyroid status was associated with ORs (95% CI) of 1.42 (1.24–1.62) for hyperuricaemia and 2.13 (1.58–2.87) for gout. Hypothyroid and hyperthyroid status was associated with population attributable risk for gout of 0.8% and 1.5% respectively.

**Table 2:** Age- and multivariate-adjusted relative risks of hyperuricaemia and gout in men with euthyroid, hypothyroid, and hyperthyroid status.

	Odds ratio (95% confidence interval)		
Risk factor	Normouricaemia	Hyperuricaemia	Gout
Thyroid function			
Euthyroid	Reference	Reference	Reference
Hypothyroid	Reference	1.08 (0.87–1.34)	1.47 (1.10–1.97)*
Hyperthyroid	Reference	0.96 (0.82–1.12)	1.37 (1.10–1.69)*
Low eGFR	Reference	4.14 (3.75–4.58)*	5.20 (4.56–5.90)*
Number of metabolic syndrome components			
0 components	Reference	Reference	Reference
1 component	Reference	1.59 (1.48–1.70)*	2.02 (1.77–2.31)*
2 components	Reference	2.51 (2.34–2.69)*	3.48 (3.05–3.97)*
3 components	Reference	3.23 (2.99–3.49)*	5.28 (4.60–6.51)*
4 components	Reference	3.37 (3.05–3.74)*	6.21 (5.27–7.31)*
5 components	Reference	3.15 (2.57–3.87)*	7.07 (5.38–9.30)*

*p<0.05; Model fit: Nagelkerke Psudo R^2^: 0.07.

**Table 3:** Age- and multivariate-adjusted relative risks of hyperuricaemia and gout in women with euthyroid, hypothyroid, and hyperthyroid status.

	Odds ratios (95% confidence interval)		
Risk factors	Normouricaemia	Hyperuricaemia	Gout
Thyroid dysfunction			
Euthyroid	Reference	Reference	Reference
Hypothyroid	Reference	1.02 (0.85–1.23)	1.44 (0.96–2.17)
Hyperthyroid	Reference	1.42 (1.24–1.62)*	2.13 (1.58–2.87)*
Low eGFR	Reference	4.29 (3.85–4.78)*	8.70 (7.10–10.67)*
Number of metabolic syndrome components			
0 component	Reference	Reference	Reference
1 component	Reference	1.91 (1.74–2.09)*	1.60 (1.20–2.13)*
2 components	Reference	3.71 (3.37–4.07)*	3.18 (2.41–4.21)*
3 components	Reference	5.53 (4.99–6.14)*	5.80 (4.35–7.72)*
4 components	Reference	7.64 (6.75–8.65)*	7.83 (5.70–10.75)*
5 components	Reference	12.15 (9.80–15.07)*	10.65 (6.50–17.45)*

*p<0.05; Model fit: Nagelkerke psudo R^2^: 0.14.

## Discussion

The present study investigated the risks of hyperuricaemia and gout associated with thyroid dysfunction in 87,813 people who received a health-screeningprogram. The correlation between TSH and serum uric acid levels was at best very weak. Although hypothyroid status was not associated with hyperuricaemia in men or women, the risk of gout increased by about 40% with hypothyroid status; however, this trend was not statistically significant in women. Hyperthyroid status was not associated hyperuricaemia in men, but in women with an OR of 1.42. Hyperthyroid status was a risk factor for gout, with ORs of 1.37 and 2.13 in men and women, respectively. In conclusion, our study showed mixed results for the association between thyroid dysfunction and hyperuricaemia. However, the risk of gout was increased in both hypothyroid and hyperthyroid individuals.

Past studies have reported an association between hyperuricaemia and thyroid disorders with conflicting results. The correlation between TSH and serum uric acid levels was weak [16], [17], which is consistent with our findings. However, the previous study has reported significantly higher prevalence of hyperuricaemia in patients with hypothyroidism [18]. In addition, thyroxin replacement in patients with hyperthyroidism was reported to reverse hyperuricaemia [18], [30]. Evidence regarding the relationship between hyperuricaemia and hyperthyroidism is even more limited. In one study, hyperuricaemia was found in 5 out of 18 hyperthyroid patients (27.7%) [18]. Another study found that patients with hyperthyroidism due to Graves' disease had significantly higher serum uric acid levels than age- and sex-matched controls [19].

In our study, the association between hypothyroid function and hyperuricaemia seems to be week. One reason for this is the consideration of renal function as a covariate in our study. Reduced renal function is a major risk factor for hyperuricemia [31], [32] since kidney is the major organ handling urate metabolism. Despite evidence suggesting links between thyroid function and hyperuricaemia [33], [34], is seems that renal function is a stronger factor determining serum uric acid levels and therefore the association between hypothyroid status and hyperuricaemia is not significant if renal function is a part of the model. It is interesting to observe hyperuricaemia in patients with hyperthyroid status, who are expected to have ahigher renal clearance of uric acid. On reason may be the increased uric acid production secondary to increased overall metabolism in hyperthyroid patients surpassing uric acid diuresis caused by hyperthyroidism [19]. Further studies should be undertaken to confirm our findings.

Consistent with previous studies [18], [23], we found a higher risk of gout in hypothyroid individuals. Interestingly, hyperthyroid status was also associated with a higher risk of gout. The weak association between thyroid status and hyperuricaemia indicated that hyperuricaemia could not fully explain the elevated risk of gout in both hypothyroid and hyperthyroid individuals. One plausible explanation is the accelerated urate crystal deposition secondary to changes in connective tissue caused by thyroid dysfunction. This hypothesis requires further study to confirm.

This study has several limitations. First, we were unable to classify patients based on the American College of Rheumatology [35] or Rome [36] classification criteria or according to the “gold standard” of urate crystal identification in synovial fluid or tophus aspirate (not commonly conducted in ordinary practice); therefore, this study may be prone to a degree of misclassification bias. Nevertheless, a previous study provided evidence for the good reliability and sensitivity of self-reported and physician-diagnosed gout [25]. In addition, we relied on a single measurement of SUA and TSH levels; therefore, we were unable to give a definite diagnosis of hyperthyroidism or hypothyroidism and cannot take into account any variation that may have occurred over time. Some medications such as diuretics may alter serum urate levels. We did not have full information on medications therefore this cannot be accounted for by our model. The strengths of this study include its use of carefully standardized methods and the large size of the study cohort. In addition, we used a cohort from a health screening, which is more representative of the general population than most previous hospital-based studies.

In conclusion, despite the mixed results for the association between thyroid status and hyperuricaemia, both hypothyroid and hyperthyroid statuses were associated with a higher risk of gout. This study suggests that a thyroid function test for gout patients may be warranted.
